# Structural and Magnetic Properties of FeNi Films and FeNi-Based Trilayers with Out-of-Plane Magnetization Component

**DOI:** 10.3390/s22218357

**Published:** 2022-10-31

**Authors:** Andrey V. Svalov, Alexandr N. Gorkovenko, Aitor Larrañaga, Mikhail N. Volochaev, Galina V. Kurlyandskaya

**Affiliations:** 1 Institute of Natural Sciences and Mathematics, Ural Federal University, 620002 Ekaterinburg, Russia; 2 Advanced Research Facilities (SGIKER), Universidad del País Vasco UPV-EHU, 48080 Bilbao, Spain; 3 Kirensky Institute of Physics FRS KSC SB RAS, 660036 Krasnoyarsk, Russia; 4 Departamento de Electricidad y Electrónica, Universidad del País Vasco UPV/EHU, 48080 Bilbao, Spain

**Keywords:** magnetic field sensors, permalloy, crystal structure, perpendicular magnetic anisotropy, stripe domains, “transcritical” state, columnar microstructure

## Abstract

FeNi films of different thickness and FeNi/(Fe, Co)/FeNi trilayers were prepared by magnetron sputtering deposition onto glass substrates. The permalloy films had a columnar microstructure. The detailed analysis of the magnetic properties based on the magnetic and magneto-optical measurements showed that at thicknesses exceeding a certain critical thickness, hysteresis loops acquire a specific shape and the coercive force of the films increase sharply. The possibility of the estimation of the perpendicular magnetic anisotropy constant using the Murayama equation for the thickness dependence of saturation field was demonstrated. The results of studies of the structural and magnetic properties of FeNi films laminated by Fe and Co spacers with different thickness are presented.

## 1. Introduction

Soft ferromagnetic films are used in various types of magnetoelectronic devices [1]. One of the ways to increase the magnetic softness of thin films is to create the layers with helical magnetic anisotropy [2]. The helical variation in the orientation of the easy magnetization axis can be obtained by the deposition of the films in the magnetic field rotating in the plane of the substrate [2,3]. A stable helical profile may occur for a film thickness greater than the specific value, defined as 2π(A/K)^1/2^, where K is the anisotropy constant, and A is the exchange stiffness constant. For FeNi films, a specific thickness depends on the deposition conditions, but it is approximately equal to 1 μm [3]. However, at a thickness of several hundreds of nanometres, FeNi films can demonstrate the transition into the so-called “transcritical” state. This state is characterized by an increase in the coercive force H_c_, appearance of the stripe domains, existence of so-called “rotatable anisotropy” and particular shape of the magnetic hysteresis loop [4,5,6,7,8,9,10,11]. An indispensable condition for the possibility of a transition is the presence of perpendicular magnetic anisotropy in the sample, while the quality factor Q = K_p_/2πM_s_, where K_p_ is the perpendicular anisotropy constant, M_s_ is the saturation magnetization of the sample, which should be less than unity. The critical thickness value L_c_ depends on the deposition conditions (deposition rate, working gas pressure) and corresponds usually to the interval of 100 to 350 nm [5,6,12,13].

The perpendicular magnetic anisotropy is responsible for the formation of the “transcritical” state in FeNi films prepared by sputtering technique. It might be a strain-caused consequence of magnetostriction and magnetoelastic contribution and/or the columnar structure of the films [5,8,14]. Laminating of the thick FeNi film with various non-magnetic and weakly magnetic spacers allows to avoid the transition into the “transcritical” state [15,16,17,18]. The use of interlayers of magnetically strong materials with a crystal structure different from that one of the Fe_20_Ni_80_ permalloy is much less studied [19,20].

Understanding the conditions of the transition into the “transcritical” state has very special importance for electronics applications as soft ferromagnetic films with magnetic permeability form part of the inductors, decoders, magnetic field sensors, and many other kinds of the devices [1,21]. Additionally, removing the “transcritical” state will make it possible to obtain soft magnetic films whose thickness will exceed the specific critical thickness mentioned above. Therefore, the condition for the preparation of the films with a helical magnetic anisotropy by the deposition of the films at the rotating magnetic field will be open. In addition, such a fundamental question as the change in the value of the perpendicular magnetic anisotropy constant K_p_ with the variation in the thickness of the FeNi films still remains open. In this work, the structural and magnetic properties of FeNi films of different thickness and FeNi/(Fe, Co)/FeNi trilayers were comparatively analyzed with the focus on the understanding of the conditions of out-of-plane magnetic anisotropy formation.

## 2. Experiment

Thin films and multilayered samples were deposited by dc magnetron sputtering onto Corning glass substrates at room temperature. Metallic circular targets of Fe_20_Ni_80_, Fe or Co were used. The deposition rates were defined by additional calibration procedure using 100 nm films of each composition. The thin films thicknesses for calibration were verified by the sharp step analyzed with Dektak 150 Stylus Profilometer (Veeco, Somerset, NJ, USA). The following deposition rates were used: 26 nm/min for FeNi layers and 1 nm/min for Fe and Co spacer materials. The thickness of the FeNi films L varied from 50 nm to 1000 nm and the thickness of Fe and Co spacers l_sp_ was subject to variations in the interval of 5 nm to 100 nm. A constant magnetic field of 250 Oe was applied parallel to the film plane during deposition in order to induce in-plane uniaxial magnetic anisotropy.

The phase analysis and average grain size was defined by X-ray diffraction technique (XRD) using a PHILIPS X’PERT PRO automatic diffractometer (Pan Analytical, Cambridge, UK) operating at 40 kV and 40 mA with Cu-Kα radiation (wavelength λ = 1.5418 Å). The configuration used for XRD studies has been theta-theta Bragg-Brentano. The particular configuration was selected as the main objective has been to irradiate the maximum sample volume to obtain the maximum signal intensity. This geometry allows the correct evaluation of the full width at half maximum (FWHM) for later size estimations. The identification of the initial phases was performed using High Score program and the powder diffraction file (PDF). The structure of the films was studied also by transmission electron microscopy (TEM) using a Hitachi HT 7700 microscope (Hitachi HT, Tokyo, Japan) and scanning electron microscope (SEM) JEOL JSM-640 (JEOL, Freising, Germany), SEM images were acquired in secondary electrons.

Magnetic measurements were carried out by means of vibrating sample magnetometer 7407 VSM vibrating-sample magnetometer (Lake Shore Cryotronics London, UK) and magneto-optical Kerr effect (MOKE) using the optical microscope Evico (Evico, Dresden, Germany).

## 3. Results and Discussion

**Figure 1:**
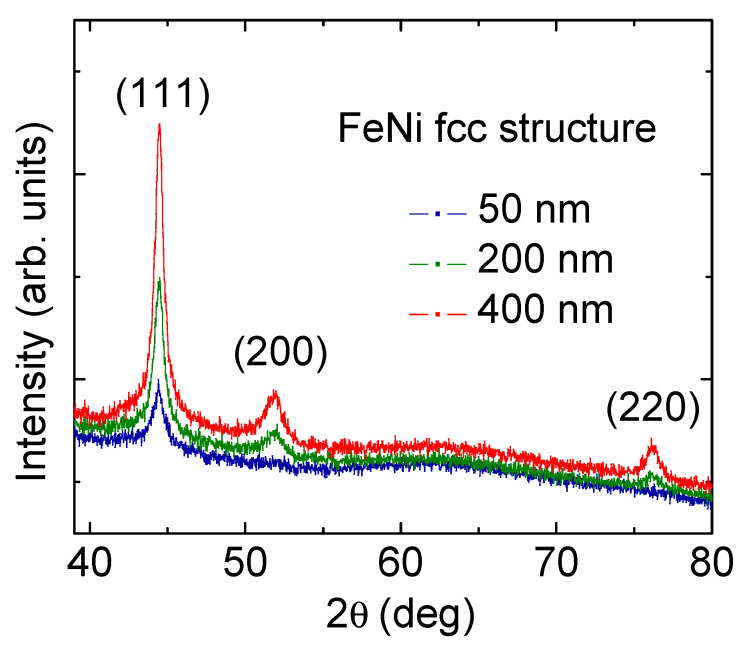
X-ray diffraction patterns for FeNi films with different thicknesses indicated in the legends. Miller indexes are shown for all bright peaks.

The X-ray diagrams for the FeNi films confirm the fcc crystalline structure of all samples under consideration (Figure 1). The presence of an (111) intense peak is a characteristic feature of the diffractograms obtained for all studied films. An increase in the film thickness is accompanied by an increase in the intensity of the (111) and (200) peaks.

For (220) peaks, an increase in the intensity is clearly observed for the films of 400 and 200 nm. However, the intensity of the (220) peak for the thinnest film of 50 nm is low and it is difficult to form a definite conclusion. This observation confirms the evidence of an improvement in crystallinity of the films. At the same time, similar average grain size of the order of 10–15 nm was observed in all cases. The size of the coherent domain was calculated using the Scherer formula for (111) reflection (2θ ≈ 44.5°). A typical cell parameter of 3.52 Å was also detected for all types of FeNi films.

**Figure 2:**
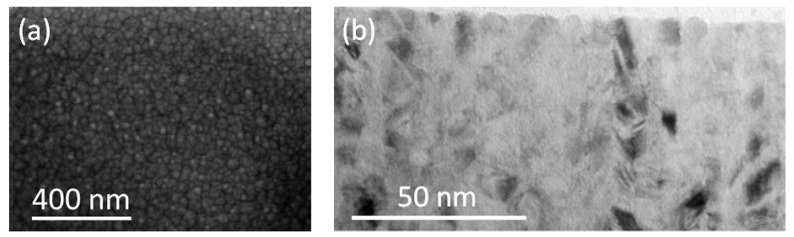
SEM morphology of the free surface (**a**) and TEM cross-sectional image (**b**) of FeNi film of 100 nm thickness.

Figure 2 shows the SEM morphology of the free surface of the FeNi film and typical example of TEM cross-sectional image of FeNi film of 100 nm. The grain-like SEM images (Figure 2a) may be due to the presence of a columnar microstructure in the film which is represented as round units in this projection. The TEM cross-sectional images confirm this assumption (Figure 2b). One can see that the geometry of the cross section of the columns on the free surface of the film is close to the circular shape and the diameters of the columns are reasonably close to the interval of 4 nm to 10 nm.

**Figure 3:**
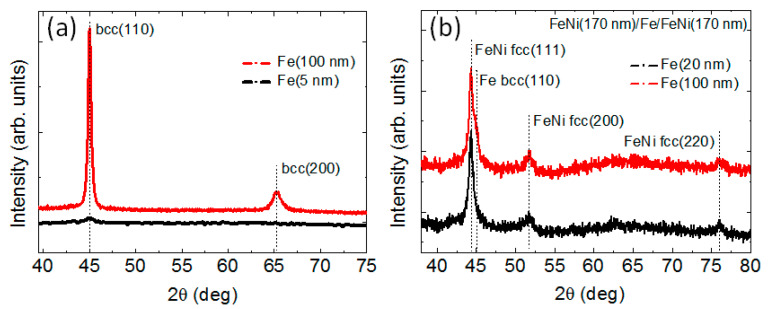
X-ray diffraction pattern for Fe films (**a**) and FeNi(170 nm)/Fe(l_Fe_)/FeNi(170 nm) trilayers (**b**).

For Fe and Co single layer films, the average grain size was approximately equal to the thickness values, but it did not exceed 15 nm. Single-layer iron films were polycrystalline bbc structures having high levels of the (110) texturing and cell parameter of 2.85 Å (Figure 3).

**Figure 4:**
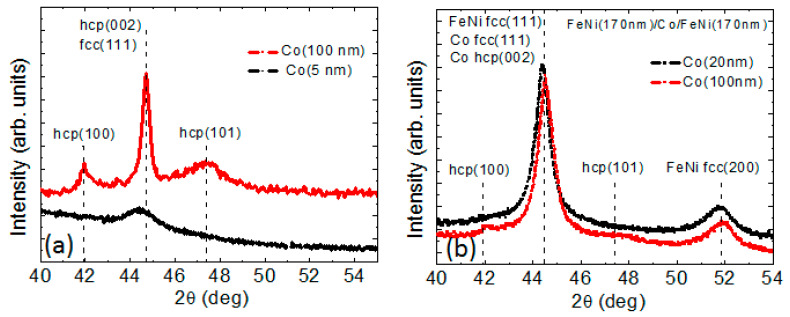
X-ray diffraction patterns for Co films of different thicknesses (**a**) and FeNi(170 nm)/Co(l_Co_)/FeNi(170 nm) trilayers (**b**). In all cases, the legends indicate the cobalt layer thickness.

The high degree of texturing observed for thin Co films did not allows us to unambiguously determine the lattice type, namely, cubic or hexagonal (Figure 4a). The metals growing incoherently on the substrates are known to prefer the close-packed orientation, like (111) plane for the fcc structure [22]. It therefore can be supposed that in thin cobalt films, both cubic and hexagonal phases can be present. The appearance of the hcp (100) and hcp (101) peaks for thicker Co films testifies the increase of the relative amount of the hexagonal phase with the increase of the Co film thickness.

**Figure 5:**
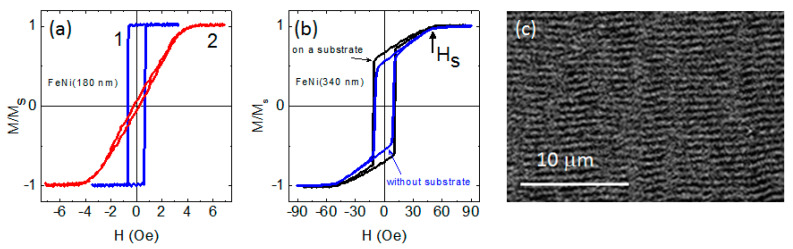
(**a**) hysteresis loops for FeNi(180 nm) film measured along (1) and perpendicular (2) to the easy axis magnetization; (**b**) hysteresis loops for FeNi (340 nm) film both in initial state and after separation from the substrate and annealing at 300 °C during one hour; (**c**) MOKE image of the stripe magnetic domain structure for FeNi (220 nm) film.

The critical thickness for FeNi films was close to 200 nm. Thinner films had uniaxial magnetic anisotropy with the anisotropy axis oriented in the sample plane (Figure 5a). The easy magnetization axis coincided with the orientation of the magnetic field applied during thin film deposition. The coercive force H_c_ of such films did not exceed 1 Oe, and the anisotropy field H_k_ was about 4 Oe.

**Figure 6:**
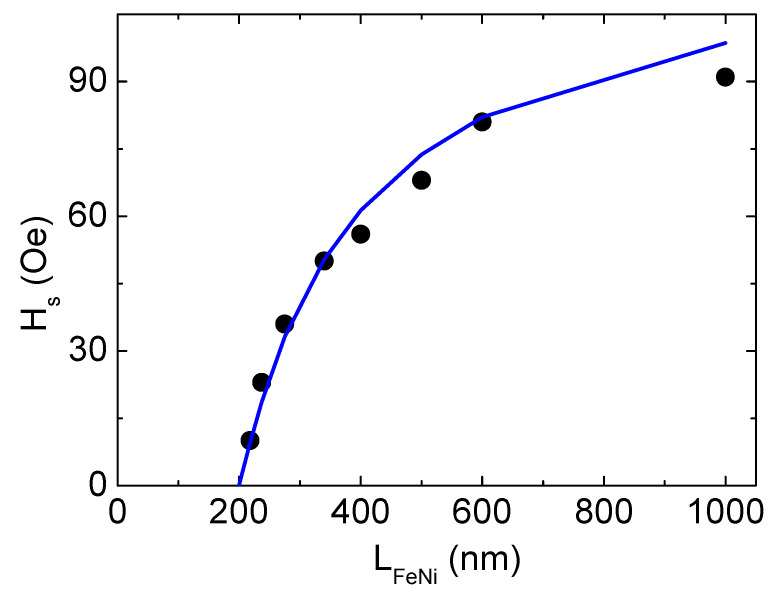
Experimental (dots) and calculated on the basis of Murayama’s model (line) thickness dependence of the saturation field H_s_ for FeNi films.

An analysis of hysteresis loops allows us to determine another important magnetic characteristic, namely, the saturation field H_s_ (Figure 5b). For the “transcritical” films [23,24,25], the relationship between the saturation field and the film parameters was previously described by Murayama’s equation [26,27]:

(1) $$1-\frac{H_{s}}{H_{k}}={\left(1+\frac{K_{p}}{2{\pi M}_{s}^{2}}\right)}^{-1/2}\frac{L_{c}}{L}$$

where H_s_ and H_k_ are the saturation and the anisotropy fields, respectively; K_p_ is perpendicular magnetic anisotropy constant; L_c_ is the critical thickness of the film. In our case, the thickness dependence of the magnetic saturation field H_s_ is well described by Murayama’s model at M_s_ = 810 G, L_c_ = 200 nm taking K_p_ value as high as 5 × 10^4^ erg/cm^3^ (Figure 6). Moreover, this result indicates that the value of the perpendicular magnetic anisotropy constant remains unchanged despite film thickness variation in the interval under consideration.

Within the framework of the Murayama model, an equation for estimation the L_c_ was also proposed:

L_c_ = 2π (A/K_p_)^1/2^ (2)

where A is the exchange stiffness constant; K_p_ is perpendicular magnetic anisotropy constant. We use the value K_p_ = 5 × 10^4^ erg/cm^3^ obtained above. Then the experimentally observed L_c_ value of 200 nm can be obtained according to expression (2) at A = 5 × 10^−7^ erg/cm. The obtained A value was in good agreement with the data reported by the other research for FeNi [28,29,30]. Thus, the observed values of L_c_ and K_p_ were in a good agreement with each other.

In order to determine the origin of perpendicular magnetic anisotropy in the studied FeNi films, the samples with low level of the internal stresses were fabricated as follows. Some films were deposited on NaCl single crystal substrate, and then they were separated from the substrate and annealed at a temperature of 300 °C during one hour. The shape of the hysteresis loops for such films was changed very little, while the values of H_c_ and H_s_ remained unchanged (Figure 5b). Thus, this result and the presence of a columnar structure (Figure 2) allow us to conclude that the source of perpendicular magnetic anisotropy in the studied FeNi films is the columnar microstructure of the films rather than the magnetostriction effect.

**Figure 7:**
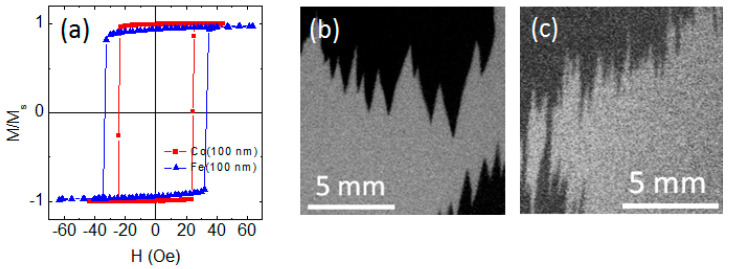
Hysteresis loops measured along easy magnetization axis for Fe (100 nm) and Co (100 nm) films (**a**). Images of the magnetic domain structure for Fe film (**b**) and Co film (**c**) obtained near H_c_ after magnetization of the samples to saturation along the easy magnetization axis (EA). The application axis of the external field is parallel to the EA and vertical sides of the images.

Fe and Co films of different thicknesses were used as magnetic spacers in three-layered FeNi/X/FeNi structures. In the present studies, the thickness of the FeNi layers was 170 nm, i.e., it was below the critical thickness of the transition into “transcritical” state L_c_ = 200 nm. In single-layered films of Fe and Co, uniaxial magnetic anisotropy was also formed in the plane of the sample under the influence of the magnetic field applied during deposition. For these films, there were no signs of transition to the “transcritical” state (Figure 7). The observed behavior is consistent with the known data for Fe films, for which the reported critical thicknesses exceed 100 nm [31]. For Co films, the reported critical thicknesses range from 10 nm to 180 nm [31,32,33]. Most likely, this distinction is due to the structural features of the films prepared under different conditions.

**Figure 8:**
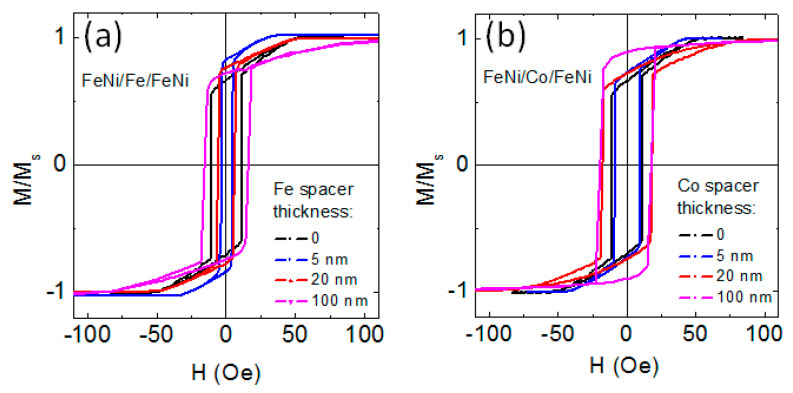
Magnetic hysteresis loops of FeNi(170 nm)/Fe/FeNi(170 nm) (**a**) and FeNi(170 nm)/Co/FeNi(170 nm) (**b**) trilayers with different thicknesses of Fe and Co spacers indicated by the legends.

The nanostructuring of thick FeNi films into trilayered structures having magnetic layers with L < L_c_ by Fe spacer does not prevent the occurrence of the “transcritical” state for FeNi/Fe/FeNi trilayers (Figure 8a). For the sample with l_Fe_ = 5 nm, this result can be related to the small thickness of the Fe spacer. For FeNi-based multilayers, the surface roughness of multilayers prepared by dc magnetron sputtering at present conditions is quite small, only a few Angstroms [34], and the thickness of the interface (the zone of mutual interlayer diffusion) is about one nm [35].

It was shown recently that for a strong/weak/strong ferromagnetic sandwich, exchange coupling between strong ferromagnetic outer layers can be reduced when the weakly ferromagnetic type spacer has a lower Curie temperature than that of the strong ferromagnetic layers [36]. However, the Curie temperature of Fe and iron-enriched interfaces surpasses the Curie temperature of the FeNi alloy. Therefore, the Fe spacer does not weaken the interlayer coupling of FeNi layers. As a result, FeNi(170 nm)/Fe/FeNi(170 nm) trilayered structure was in the “transcritical” state like a single layered FeNi(340 nm) film. As l_Fe_ increases, the Fe interlayer acquires more and more structural independence, and its magnetic characteristics become closer to those of bulk iron.

Let us estimate the possible value of the perpendicular magnetic anisotropy constant in the Fe films under consideration. According to the results of structural studies, the Fe films had the (110) texture. If one takes into account only the first magnetocrystalline anisotropy constant and neglects the magnetoelastic contribution, then for a cubic iron film with (110) texture, the effective perpendicular anisotropy constant can be determined as K_p_ = 3/16 × K_1_ [37]. For iron, the magnetocrystalline anisotropy constant K_1_ = 4.8 × 10^5^ erg/cm^3^ [35], then K_p_ = 9 × 10^4^ erg/cm^3^.

Due to the strong interlayer interaction, the FeNi/Fe/FeNi three-layer system behaves as a whole. Based on this fact, let us make an approximating estimate of the L_c_ value for the FeNi(170 nm)/Fe(100 nm)/FeNi(170 nm) sample using expression (2). Being aware of the largely arbitrary nature of the procedure, let us average the values of K_p_ and constant A using the corresponding values for FeNi films (K_p_ = 5 × 10^4^ erg/cm^3^, A = 0.5 × 10^−6^ erg/cm) and Fe (K_p_ = 9 × 10^4^ erg/cm^3^, A = 2.1 × 10^−6^ erg/cm) and taking into account the ratio of the layer thicknesses. As a result, for FeNi(170 nm)/Fe(100 nm)/FeNi(170 nm) we obtain K_p_ = 5.9 × 10^4^ erg/cm^3^, A = 0.9 × 10^−6^ erg/cm and L_c_ = 240 nm. The resulting estimate agrees with the experimentally observed “transcritical” state for FeNi(170 nm)/Fe(100 nm)/FeNi(170 nm) film.

Laminating of the thick FeNi film with cobalt spacer allows to avoid the transition into the “transcritical” state at l_Co_ > 50 nm. For small values of l_Co_, the situation is apparently the same as for three-layered samples with small l_Fe_. The results of structural studies indicate the possibility of the presence of both fcc (texture (111)) and hcp (possible texture (002)) cobalt phases in the layers under consideration. Both of these crystallographic orientations contribute to the appearance of perpendicular magnetic anisotropy in the film, since the [111] axis of the fcc lattice and the [002] axis of the hcp lattice are easy magnetization axes related to magnetocrystalline anisotropy. However, the uncertainty in the number of hcp and fcc crystalline phases in thick Co layers makes it meaningless to try to estimate the value of the effective constant of perpendicular magnetic anisotropy in Co interlayers. In addition, the value and sign of this constant can be affected by a magnetoelastic contribution, the evaluation of which is beyond the scope of the present study, but which can be significant in Co films [38]. Here, for now, we only indicate the possibility to avoid the transition into the “transcritical” state by means of laminating of the thick FeNi film with Co spacers. Finding out the exact reason for this requires a separate thorough investigation. In addition, laminating of the thick FeNi film with cobalt spacer does not reduce the coercive force of FeNi/Co/FeNi trilayers to the level of the coercive force of single-layer soft magnetic films FeNi with L < L_c_, as happens in the case of non-magnetic spacers [15,16,17,18]. Nevertheless, the absence of the “transcritical” state and, as a consequence, the absence of rotatable anisotropy opens the possibility of more precise control of the features of the magnetic anisotropy of such film systems. This creates a perspective for the preparation of the films with a helical magnetic anisotropy by the deposition of the films at the rotating magnetic field and possible manipulation of the magnetization of helimagnets through the spin-transfer torque effect [39].

Magnetically soft thin FeNi films and FeNi films based multilayered structures are widely used in sensor applications. In many cases, they are used as the main materials of sensitive element for low magnetic field measurements [40,41,42]. However, for the functionality of the magnetic field sensors they must be integrated in the electronic circuit with compatible characteristics, for example inductor structures with magnetic films, including FeNi components [43]. In addition, present day applications request multifunctional devices in which positioning plays very important role. With respect, soft magnetic films may contribute as additional positioning element or elements array up to flexible devices [44,45,46,47]. One can also mention the need of electromagnetic protection of electronic devices including magnetic sensors for correct operation and avoiding the unwanted interferences for which FeNi films with high magnetic permeability and especially FeNi-based multilayers with tunable dynamic magnetic properties are quite suitable [48,49,50].

## 4. Conclusions

Structural and magnetic properties of FeNi films of different thickness and FeNi/(Fe, Co)/FeNi trilayers were comparatively analyzed. It was found that the permalloy films had a columnar microstructure. It is shown that perpendicular magnetic anisotropy constant does not depend on the FeNi film thickness in the range of the thicknesses of 50 to 1000 nm. Based on the results of structural and magnetic measurements, it can be concluded that the main origin of the perpendicular magnetic anisotropy in FeNi films is the columnar microstructure. It was demonstrated that for FeNi(170 nm)/Fe/FeNi(170 nm) trilayers the “trascritical” state exists at any Fe spacer thickness up to 100 nm. At the same time, for FeNi(170 nm)/Co/FeNi(170 nm) trilayers the “transcritical” state disappears at l_Co_ > 50 nm. It is possible to avoid the transition into the “transcritical” state by means of laminating of the thick FeNi film with Co spacers.

## Data Availability

Data available from the corresponding author upon reasonable request.
